# The Neuroprotective Lipocalin Apolipoprotein D Stably Interacts with Specific Subtypes of Detergent-Resistant Membrane Domains in a Basigin-Independent Manner

**DOI:** 10.1007/s12035-022-02829-z

**Published:** 2022-04-22

**Authors:** Miriam Corraliza-Gomez, Manuela del Caño-Espinel, Diego Sanchez, Maria D. Ganfornina

**Affiliations:** Instituto de Biología y Genética Molecular, Unidad de Excelencia, Universidad de Valladolid-CSIC, 47003 Valladolid, Spain

**Keywords:** Neuroprotection, Lipid rafts, Plasma membrane, Lysosome, Lipid peroxidation, Endocytosis

## Abstract

Accumulated evidence points to the lipocalin apolipoprotein D (ApoD), one of the few genes consistently upregulated upon brain ageing and neurodegeneration, as an endogenous controller of the redox state of cellular and extracellular lipid structures. This biochemical function has downstream consequences as apparently varied as control of glycocalyx and myelin compaction, cell viability upon oxidative stress or modulation of signalling pathways. In spite of this knowledge, it is still unclear if ApoD function requires canonical receptor-mediated transductions systems. This work aims to examine ApoD-cell membrane interaction and its dependence on a proposed ApoD receptor, Basigin. Whole and fractionated membrane preparations from the brain, primary astrocytes, glial and neuronal cell lines, reveal ApoD as a very specific component of particular subtypes of detergent-resistant microdomains (DRMs). ApoD interacts in vitro with neuronal membranes and is stably associated with astrocytic membranes. ApoD associates with DRMs with specific buoyancy properties that co-fractionate with plasma or late-endosome-lysosome markers. A mass spectrometry analysis reveals that these Triton X-114 DRMs contain both plasma membrane and endosomal-lysosomal compartment lipid raft proteins. ApoD-DRM association is maintained under metabolic and acute oxidative stress conditions. However, ApoD-membrane interaction, its internalization and its lipid-antioxidant function do not require the presence of Basigin. This work supports a stable association of ApoD with membranes, independent of Basigin, and provides the basis to fully understand ApoD antioxidant neuroprotective mechanism as a mechanism taking place in specific membrane subdomains.

**Supplementary Information:**

The online version contains supplementary material available at 10.1007/s12035-022-02829-z.

## Introduction

Cellular membranes are essential for life. They separate the intra and extracellular media, define compartments within the cell and represent a selective barrier at the same time that a site for communication and transduction of biological messages between and within cells. Mechanisms for membrane repair were required early in evolution [1], quite importantly since the origin of the eukaryotic cell with its complex set of membranous compartments. If keeping cellular membranes in check is important for any living cell, it is of special relevance for those constituting our nervous system, where most neurons are long-lived cells, and the different cell types possess highly specialized functional membrane domains. Upon injury, membrane repair mechanisms are a priority at both the plasma and lysosomal membranes, whose damage would put potentially adverse extracellular environment in contact with the cell interior or would jeopardize optimal lysosomal enzymatic activities.

Both the plasma and late-endosome-lysosome membranes have specialized liquid-ordered domains (detergent-resistant membrane domains, DRMs; also known as lipid rafts), rich in sphingomyelin, cholesterol and gangliosides [2]. Protein-lipid interactions in DRMs conform a “macromolecular landscape” bringing together partners of complex signalling and functional interactions.

The lipid-binding protein apolipoprotein D (ApoD), first discovered as part of HDL_3_ particles purified from human plasma [3], is a member of the lipocalin family, which comprise mainly extracellular secreted proteins [4]. ApoD is one of the few genes consistently upregulated in the vertebrate ageing brain [5] and in an amazingly wide array of neurodegenerative and psychiatric diseases of diverse aetiology, including Alzheimer’s disease or lysosomal storage diseases (e.g. [6, 7]). A recent systematic review on the apparently diverse functions carried out by ApoD within and outside the nervous system [8] supports a unique biochemical role: the management of cellular and extracellular lipid structures and their redox state. Two recent discoveries have triggered a complete change of paradigm, transforming our view of this finely regulated and evolutionary conserved lipid-binding protein. First, ApoD is able to reduce free radical-generating lipid hydroperoxides to inert lipid hydroxides. This antioxidant activity depends on a particular methionine residue, at the rim of the lipid binding pocket, and has been demonstrated with oxidized lipids in solution or auto-oxidized liposomes [9]. Furthermore, antioxidant activity correlates with ApoD function in different tissue contexts [10, 11]. Second, ApoD, expressed by glial cells and secreted to the extracellular milieu (on exosomes or lipoparticles), is internalized by glia and neuronal cells and targeted in a finely controlled way to the subset of lysosomes most sensitive to oxidative stress (OS). The stable presence of ApoD in lysosomes is sufficient and necessary for the recovery from oxidation-induced lysosomal membrane permeabilization [12]. This property determines the viability of cells under experimental OS [13], in pathologies hampering lysosomal function like Niemann-Pick type A disease [6] or in proteinopathies requiring optimal autophagic activity [14]. Furthermore, lysosomal ApoD is required for an adequate plasma membrane-lysosome traffic that controls the glycocalyx composition, particularly ganglioside content and distribution, with relevant functional consequences in the nervous system: Without ApoD, complete myelin compaction is halted throughout life [15].

Predictions for direct interaction of ApoD with cellular membranes can be formulated based on its association with lipoprotein particles [3] and liposomes [9], as well as on ApoD downstream effects on lipid peroxidation studied at organismal, cellular and molecular levels [9, 13, 16]. This property is evidenced by ApoD immunoreactivity at the plasma or intracellular organelle membranes, first observed by Boyles et al. [17] and recently demonstrated by immunogold electron microscopy ([12, 18], reviewed by [8]). Nevertheless, in spite of all the advancements in the understanding of ApoD biology and functional consequences for brain health and disease, we still need to elucidate how ApoD interacts with relevant cellular membranes if we want to understand its mechanism of action at the molecular level.

Additionally, ApoD has demonstrated downstream effects on particular signalling cascades (e.g. PI3K-Akt pathway) [11]. However, whether these are mediated by a canonical receptor-mediated transduction mechanism is still unknown. Candidate ApoD receptors have been postulated from experiments where downstream consequences of ApoD exposure are modified by antagonists of some receptors (LDLR, CXCR-4) [19], or by biochemical approaches combining classic two-hybrid systems with co-immunoprecipitation experiments. The later approach has reported two transmembrane glycoproteins (CD147/Basigin (BSG) [20] and the Scavenger receptor class B type 1 (SRB1) [21]) and an extracellular glycoprotein, osteopontin (OPN) [22], as candidate ApoD protein interactors (reviewed by [8]). However, two independent evidences indicate that ApoD is able to interact with lipidic structures without requiring a protein–protein contact: (i) the direct antioxidant action of ApoD on phospholipid vesicles [9], and (ii) that ApoD-LDL interaction is hampered in the presence of detergent, and requires the presence of the hydrophobic surfaces on the protein [23]. Since unilamellar vesicles represent a simplified version of the outer phospholipid layer of HDLs, LDLs or a membrane bilayer, these data suggest that ApoD might not require a proteic membrane receptor. In this scenario, ApoD downstream effects might depend on its ability to modulate the lipid context of signalling elements, as it is the case for insulin pathway inhibition by NLaz, an ApoD Drosophila homologue, which depends on its ability to modify PI3K association with the plasma membrane [24].

Studying the interaction of ApoD with cellular membranes is therefore mandatory in order to understand its neuroprotective functions. In this work, we demonstrate that: (1) ApoD is stably associated with cellular membranes of astroglial cells and interacts with membranes of neurons that do not express ApoD; (2) ApoD associates with a particular subset of DRMs with distinctive buoyancy properties, and co-fractionates with both plasma and late-endosome-lysosome membrane markers; (3) ApoD-membrane association is stable under metabolic and OS conditions; and (4) ApoD-membrane interaction, its internalization and its lipid-antioxidant function do not require the presence of the candidate ApoD protein receptor BSG.

## Methods

### Animals, Cell Cultures and Treatments

C57BL/6 J mice were maintained in positive pressure-ventilated racks at 25 ± 1 °C with 12-h light/dark cycle, fed ad libitum with standard rodent pellet diet (Global Diet 2014; Harlan Inc., Indianapolis, IN, USA) and allowed free access to filtered and UV-irradiated water. Experimental procedures were approved by the University of Valladolid Animal Care and Use Committee, following the regulations of the Care and the Use of Mammals in Research (European Commission Directive 86/609/CEE, Spanish Royal Decree 1201/2005).

Human astrocytoma 1321N1 cell line (ECACC-86030402) was cultured at 37 °C in a humidity-saturated atmosphere containing 5% CO_2_ in Dulbecco-modified Eagle’s medium (DMEM; Lonza) supplemented with heat-inactivated 5% foetal bovine serum (FBS), 1% L-glutamine and 1% penicillin/streptomycin (PS). Human neuroblastoma SH-SY5Y cells (ATCC CRL-2266) were cultured as above, but in DMEM supplemented with 4.5 g/l glucose, heat-inactivated 10% FBS, 1% L-glutamine, 1% PS and 1% nonessential amino acids supplement (Lonza). Human glioblastoma U87 WT and Bsg-KO (clone #8) cell lines [25] were kindly supplied by Dr. J. Pouysségur (Univ. Nice), and cultured as indicated for the 1321N1 line. They were generated by Zinc Finger Nucleases-Mediated Targeted Genome Editing, with Bsg-KO incorporating cytoplasmic GFP as reporter gene. Primary cortical astrocytes from 0 to 1-day-old mice were cultured as described [13].

Exogenous Addition of ApoD.

Human ApoD (hApoD) purified from breast cystic fluid [26] was added (10–100 nM) to the cell cultures for 2–3 h. Fluorescence labelling of purified hApoD was carried out with the Alexa-labelling kit (Invitrogen), following manufacturer specifications.

Paraquat (PQ) Treatment.

Cells were cultured in phenol red-free DMEM supplemented with 1% L-glutamine, 1% PS and 0.2% charcoal-stripped FBS (to minimize the presence of antioxidants in media while partially maintaining serum growth factors). This medium without additives was used as our low-serum (LS) condition, representing a mild metabolic stress. Cells were treated for 2–24 h with PQ (500 μM) prepared in LS medium.

### Crude Membrane Preparation

Tissues or cell pellets were homogenized in a Potter glass homogenizer on ice in TNE (Tris 50 mM pH 7.4, NaCl 150 mM, EDTA 5 mM) with protease inhibitor cocktail (PI; Roche) and centrifuged at low-speed (3000 g, 10 min). The supernatant was ultracentrifuged in a Beckman Optimal-100XP (100 Ti rotor, 100,000 g, 75 min), and the membrane pellets were resuspended in TNE + PI. Protein concentration was quantified using Micro-BCA procedure (Pierce).

### In vitro Membrane-Binding Analysis

Membranes resuspended in TNE + PI were divided in 3 aliquots and bath-sonicated for 3 min. Purified proteins (hApoD, 10–100 nM; BSA 100 nM) were added and membranes incubated for 30 min (20 °C, 700 rpm; Eppendorf Thermomixer). Membranes were pelleted by ultracentrifugation as above. Supernatant was collected (un-bound fraction) and concentrated (Amicon Ultra-4, Ultracel-10 K), while membrane pellet (bound fraction) was resuspended in lysis buffer (10 mM HEPES pH 7.6, 100 mM KCl, 1 mM EDTA, 1% sodium deoxycholate, 1% NP-40, 0.1% SDS, 10% glycerol, 1 mM DTT, 1X PI). Equivalent volumes of membrane and supernatant fractions were loaded for immunoblot analysis. Net bound or un-bound ApoD (pmols) per mg of membrane proteins was estimated from the relative signals (membrane vs. supernatant) detected in each blot. 

### Isolation of DRMs by Sucrose Multistep-Gradient Centrifugation

Membrane separation in detergent-soluble and resistant fractions was performed as described by Franco-Villanueva et al. [27] with some modifications. Briefly, 300 µg of membrane proteins was incubated in 1% Triton X-114 (TX114) in TNE + PI for 40 min at 4 °C in a rotating mixer, and was then diluted with a sucrose-TNE-PI solution to achieve a final 55% sucrose concentration. Different concentrations of sucrose-TNE-PI were layered on the solubilized membranes, depending on the step gradient design. The gradients were ultracentrifuged at 4 °C (Beckman Optimal-100XP; SW 40 rotor (100,000 g, 20 h)) after which 12–13 fractions were collected top to bottom. Each gradient fraction was incubated in 40% trichloroacetic acid (TCA) for 20 min. Protein precipitates were washed twice with cold ethanol and centrifuged, and the dried samples were resuspended in sample buffer for subsequent discontinuous SDS-PAGE and immunoblot analysis.

### Cholesterol and Sphingomyelin Quantification

Cholesterol and sphingomyelin were quantitated by fluorimetric assays in individual sucrose-gradient fractions. For cholesterol, we used the Amplex Red Assay kit (Invitrogen) following manufacturer specifications and measuring fluorescence with *λ*_*Ex*_ = 535 and *λ*_*Em*_ = 590. For sphingomyelin, we used a protocol described by Arroyo et al. [28], where fluorescence derived from sphingomyelin degradation is measured with *λ*_*Ex*_ = 327 and *λ*_*Em*_ = 420.

### Immunoblot Analysis

Cell lysates, membrane preparations, concentrated supernatants or sucrose-gradient fractions were analysed by immunoblot under denaturing and reducing conditions (0.5% SDS, 25 mM DTT). After electrophoresis, proteins were transferred to PVDF membranes using standard procedures, and exposed to rabbit serum anti-hApoD (custom made by Abyntek Biopharma against purified ApoD [26]), goat serum anti-mouse ApoD (sc-34760; Santa Cruz Biotechnology), mouse anti-Basigin (MA1-19,201; Thermo Scientific), mouse anti-caveolin-1 (sc-894; Santa Cruz Biotechnology), mouse anti-Lamp2 (H4B4; DSHB), mouse anti-flotillin 1 (610,820; Becton Dickinson) or mouse anti-PMCA (sc-20028; Santa Cruz Biotechnology), followed by HRP-conjugated secondary antibodies (Santa Cruz Biotechnology). The rabbit serum anti-hApoD specificity has been validated in our previous reports [6, 12] by comparison with other anti-hApoD sera or monoclonal antibodies. In this work, the serum was purified with gel Melon (44,600; Thermo Scientific-Pierce) and titrated through dilution series for each technique using the astrocytic (1321N1) and neuronal (SH-SY5Y) cell lines as positive and negative controls respectively. No cross-reactivity with mouse ApoD has been detected. The goat serum anti-mouse ApoD has been also validated and titrated in our previous report [6] using ApoD-KO tissues as negative control. Membranes were developed with ECL reagents (Millipore) and the signal recorded with a digital camera (VersaDoc; BioRad). The integrated optical density of the immunoreactive protein bands was measured in images taken within the linear range of the camera, avoiding signal saturation.

### Proteomics Analysis

Proteomics analysis was performed at the Proteomic Service of the Spanish National Centre for Biotechnology (CNB-CSIC, http://proteo.cnb.csic.es/proteomica/). Protein samples (5–10 µg) of TX114-DRMs obtained from membranes of mouse primary astrocytes (four independent preparations), and floating over the 35% sucrose layer, were dissolved in 8 M urea and 25 mM ammonium bicarbonate, reduced and alkylated with iodoacetamide [29], digested by incubation overnight at 37 °C with Trypsin (Sigma-Aldrich; ratio 25:1) and desalted with ZipTip (Merck). The digested peptides (2 µg) were extracted and subjected to 1D-nano LC ESI-MSMS analysis using a nano liquid chromatography system (Eksigent Technologies nanoLC Ultra 1D plus, SCIEX, Foster City, CA) coupled to a high-speed Triple TOF 5600 mass spectrometer (SCIEX, Foster City, CA) with a Nanospray III source. A silica-based reversed phase Acquity UPLC® M-Class Peptide BEH C18 analytical column (75 µm × 150 mm, 1.7 µm particle size and 130 Å pore size; Waters) was used. The trap column was a C18 Acclaim PepMapTM 100 (100 µm × 2 cm, 5 µm particle diameter, 100 Å pore size; Thermo Scientific), switched on-line with the analytical column. The loading pump delivered a solution of 0.1% formic acid in water at 2 µl/min. The nano-pump provided a flow rate of 250 nl/min and was operated under gradient elution conditions. Peptides were separated using a 150-min gradient ranging 2–90% mobile phase B (mobile phase A: 2% acetonitrile, 0.1% formic acid; mobile phase B: 100% acetonitrile, 0.1% formic acid). Injection volume was 5 µl.

Data acquisition was performed with a TripleTOF 5600 System (SCIEX, Foster City, CA), acquired using an ionspray voltage floating (ISVF) 2300 V, curtain gas (CUR) 35, interface heater temperature (IHT) 150, ion source gas 1 (GS1) 25 and declustering potential (DP) 100 V. All data were acquired using information-dependent acquisition (IDA) mode with the Analyst TF 1.7 software (SCIEX, USA). For IDA parameters, 0.25-s MS survey scans (mass range of 350–1250 Da) were followed by 35 MS/MS scans of 100 ms (mass range of 100–1800, total cycle time was 4 s). Switching criteria were ion m/z greater than 350 and smaller than 1250, with charge state of 2–5 and an abundance threshold of more than 90 counts (cps). Former target ions were excluded for 15 s. IDA rolling collision energy (CE) parameter script was used for automatically controlling the CE.

Data analysis and label-free quantification were processed using the PeakView® 2.2 software (SCIEX, Foster City, CA) and exported as mgf files. Proteomics data analysis was performed using 4 different search engines (Mascot Server v.2.6.1, OMSSA, X!Tandem and Myrimatch) and a target/decoy database built from sequences in the *Mus musculus* reference proteome at Uniprot Knowledgebase.

Gene ontology enrichment analysis and functional annotation of proteins recovered from TX114-DRMs of mouse primary astrocytes were performed using DAVID 2021 (https://david.ncifcrf.gov/home.jsp). Comparisons of our protein list with mouse raft proteins were performed in the RaftProtV2 database (https://www.raftprot.org/).

### Quantitative RT-PCR

Total RNA was extracted with QIAzol (Qiagen), its concentration measured with a Nanodrop spectrophotometer and its quality assessed by agarose electrophoresis. Following DNAse treatment, 500 ng of total RNA was reverse-transcribed with PrimeScript (Takara Bio Inc., Otsu, Japan) using Oligo-dT primers and random hexamers. The resulting cDNA was used as a template for quantitative RT-PCR (RT-qPCR) using SybrGreen (SYBR® Premix Ex Taq™ kit, Takara). The primers used for RT-qPCR are hApoD-forward (5′-CCACCCCAGTTAACCTCACA), hApoD-reverse (5′-CCACTGTTTCTGGAGGGAGA), human RPL18-forward (5′-CCATCATGGGAGTGGACAT) and RPL18-reverse (5′-CACGGCC GTCTTGTTTTC).

Amplifications were performed in 5 (ApoD) or 4 (RPL18) replicates in a Rotor-Gene RG-3000 (Corbett Research, UK) thermal cycler. Cycling conditions were 95 °C, 5 min; 40 cycles (95 °C, 30 s; 55 °C, 15 s; 72 °C, 15 s). Transcription levels were assessed with the ΔΔCT method [30] using normalization to Rpl18 for each condition. Statistically significant differences of transcriptional changes were evaluated with a Mann–Whitney *U* test [31] using ΔCT of each replica (calculated by subtracting the average CT of the reference gene for each sample).

### Immunocytochemistry, Image Acquisition and Analysis

Cells attached to poly-L-lysine-treated coverslips were fixed with 4% formaldehyde for 10 min at 20 °C. Following washes in PBS, cells were blocked and permeabilized with Tween-20 (0.1%) and 1% non-immune (goat or cow) serum. Cells were incubated with primary antibodies: mouse anti-Basigin (MA1-19,201; Thermo Scientific), mouse monoclonal anti-Lamp2 (H4B4; DSHB), goat anti-4-hydroxynonenal (HNE12-S; Alpha Diagnostic) or rabbit serum anti-hApoD. All antibodies were prepared in blocking solution. Alexa Fluor® 594/488-conjugated IgGs (Jackson Labs) were used as secondary antibodies. After washes in PBS, cells were mounted in EverBrite™ mounting medium with DAPI and sealed with CoverGrip™ (Biotium).

Confocal images were obtained with a 63 × oil immersion objective (HCX PL Apo CS NA = 1.4; Leica) attached to a confocal DMI 6000B microscope with a TCS SP5 confocal system (Leica) equipped with AOBS and AOTF systems. Fluorophores were excited with WLL laser and a 405 line controlled by the LAS AF software (Leica). Emissions were collected with the AOBS system and three spectral detectors. Laser power and detection gains were set by scanning control samples labelled with secondary antibody alone. We ensured to obtain similar dynamic ranges in our images, and adjusted gain and offset using LUTs. In this manner, bleed through can be neglected. Negative control images showed very weak and homogeneous background. We obtained confocal sections under constant conditions to minimize image acquisition variation. Images were stored as 1024 × 1024 pixels and 8-bit TIFF files.

Z-series (xyz scan) were performed with an optimal value of the steps size calculated for the wavelength used to fulfil the Nyquist theorem. The optical section thickness was 0.772 µm. Pixel size corresponded to 0.06 × 0.06 μm^2^, and scanning performed with 1.0 Airy unit pinhole size. Images were processed and analysed with the FIJI software.

### MTT Viability Assay

Net cell culture-reducing activity was measured using the MTT (3-(4,5-dimethylthiazol-2-yl)-2,5-diphenyltetrazolium bromide) colorimetric assay as a global approximation to cell viability, as previously described [32]. Briefly, after MTT exposure for 3 h, cells were incubated in isopropanol with 10% Triton X-100, and the solubilized formazan was measured by spectrophotometry using the SOFTmax Pro microplate reader (Molecular Devices). Absorbance was measured at *λ* = 570 nm after subtracting the *λ* = 690 nm background.

### Statistical Analysis

Statistical analyses were performed with the SPSS v.19 (IBM) and SigmaPlot v.11.0 (Systat) software. A *p*-value < 0.05 was set as a threshold for significant changes. The tests used for each experiment are stated in figure legends.

## Results

### Native ApoD is Associated with Detergent-Resistant Domains in Membranes of Human and Mouse Glial Cells

Our current knowledge predicts that ApoD influence on cellular (plasma or lysosomal) membranes must be directly linked to its neuroprotective function. In this work, we study ApoD-membrane interaction at the biochemical level, using ApoD-expressing cells or brain tissue, as well as exogenous addition of ApoD to cells or membrane preparations.

**Fig. 1:**
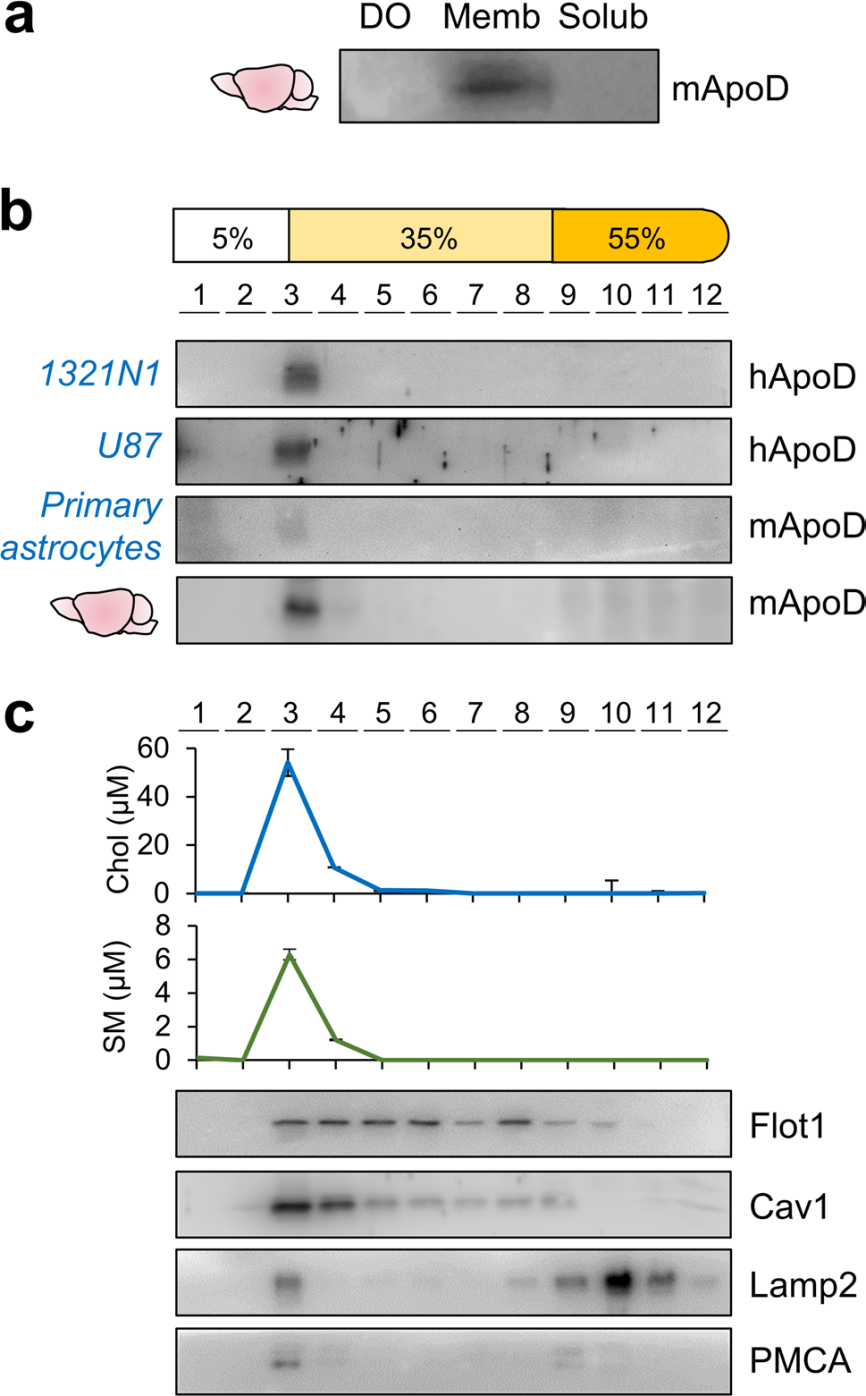
ApoD is associated with detergent-resistant domains in membranes of human and mouse glial cells. **a** Immunoblot analysis of ApoD upon centrifugal fractionation of mouse brain cells in dense organelles (DO) and membrane or soluble fractions. **b** Immunoblot analysis of ApoD in Triton X-114-solubilized membrane fractions of several cell preparations fractionated in 12 samples by a 5–35-55% discontinuous sucrose density centrifugation. Both human and mouse ApoD associate almost exclusively with the DRMs, floating over the 35% sucrose layer (fraction 3). Representative immunoblots are shown (1321N1 cells, *n* = 11 independent experiments; U87 cells, *n* = 3; primary mouse astrocytes, *n* = 3; mouse brains, *n* = 4). **c** DRM fraction 3 from mouse brain preparations is enriched in cholesterol and sphingomyelin (*n* = 6 mice; 3 males and 3 females; average ± SEM shown; no difference between sex was detected). The membrane proteins Flot1, Cav1, Lamp2 and PMCA also appear in fraction 3, but show distinct and variable mobility in the sucrose gradient

Figure 1a shows a clear association of native ApoD with membranes extracted from mouse brains. No signal was detected either in the first low-speed pellet containing dense organelles, or in the supernatant after ultracentrifugation of cell membranes. To test whether this ApoD-membrane association takes place in particular membrane microdomains, we fractionated membrane preparations after cold solubilisation with TX114. Due to its differential ability to dissolve outer membrane vs. inner membrane proteins (analysed in prokaryotes [33]), DRMs obtained after TX114 solubilisation are expected to be enriched in lipid rafts originated in the endolysosomal compartment.

To test this, we performed a proteomic analysis of TX114-DRMs (fraction 3) from primary murine astrocytes (Online Resource 1a-c and Online Resource 4). We found that the 168 proteins identified in our samples show a significant GO enrichment in plasma membrane and membranes from the endolysosomal compartment. Also, a comparison with the ProtRaftV2 database revealed that 71% of proteins from our samples appear in the annotated proteome of mouse membrane rafts (1165 proteins). Moreover, 21 and 17 proteins show cell compartment GO tags for membrane rafts and lysosomes respectively (Online Resource 1c). These results confirm that membrane raft proteins are abundant in TX114-DRMs of murine primary astrocytes. Of special interest is the GO enrichment of lysosomal proteins when comparing our DRMs (10.2% of the identified proteins) with the mouse raft proteins in the RaftProtV2 database (4.9%). This is coherent with the fact that only one, out of 138 mouse experiments included in RaftProtV2, uses TX114. Our results thus show that TX114-DRMs in eukaryotic cells are enriched in membranes from the endolysosomal compartment.

ApoD protein was detected almost exclusively in the TX114-DRM (fraction 3), floating over the 35% sucrose layer (Fig. 1b), of membranes isolated from different sources: two human astrocytic cell lines (1321N1 astrocytoma and U87 glioblastoma), primary mouse astrocytes and mouse brain. Characterization of the discontinuous sucrose gradients (Fig. 1c) reveals the expected cholesterol and sphingomyelin enrichment of these membrane fractions. The presence of the late-endosome-lysosome marker Lamp2 and the plasma membrane calcium ATPase (PMCA) in the ApoD-positive DRM fraction confirms that it contains both lysosomal and plasma membranes, in agreement with our mass spectrometry analysis (Online Resource 1a-c and 4) and with ApoD subcellular localization in glial cells [12, 15]. Lamp2 and PMCA, however, are present in both DRMs and detergent-soluble fractions. The presence of DRM markers Flot1 and Cav1 in floating membrane domains with variable mobility up the sucrose step gradient, often reported [27, 34], is also indicative of their presence in diverse types of membrane domains with different physicochemical properties (see quantification of protein profiles in Online Resource 1d-f). In contrast, the almost exclusive detection of ApoD in the fraction floating over 35% sucrose (with a small proportion present in TX114-soluble fractions) places ApoD as a robust marker for specific types of DRMs with high buoyancy properties.

### ApoD is Stably Present in DRMs upon Acute Oxidative Stress Exposure, Unlike its Putative Receptor BSG

**Fig. 2:**
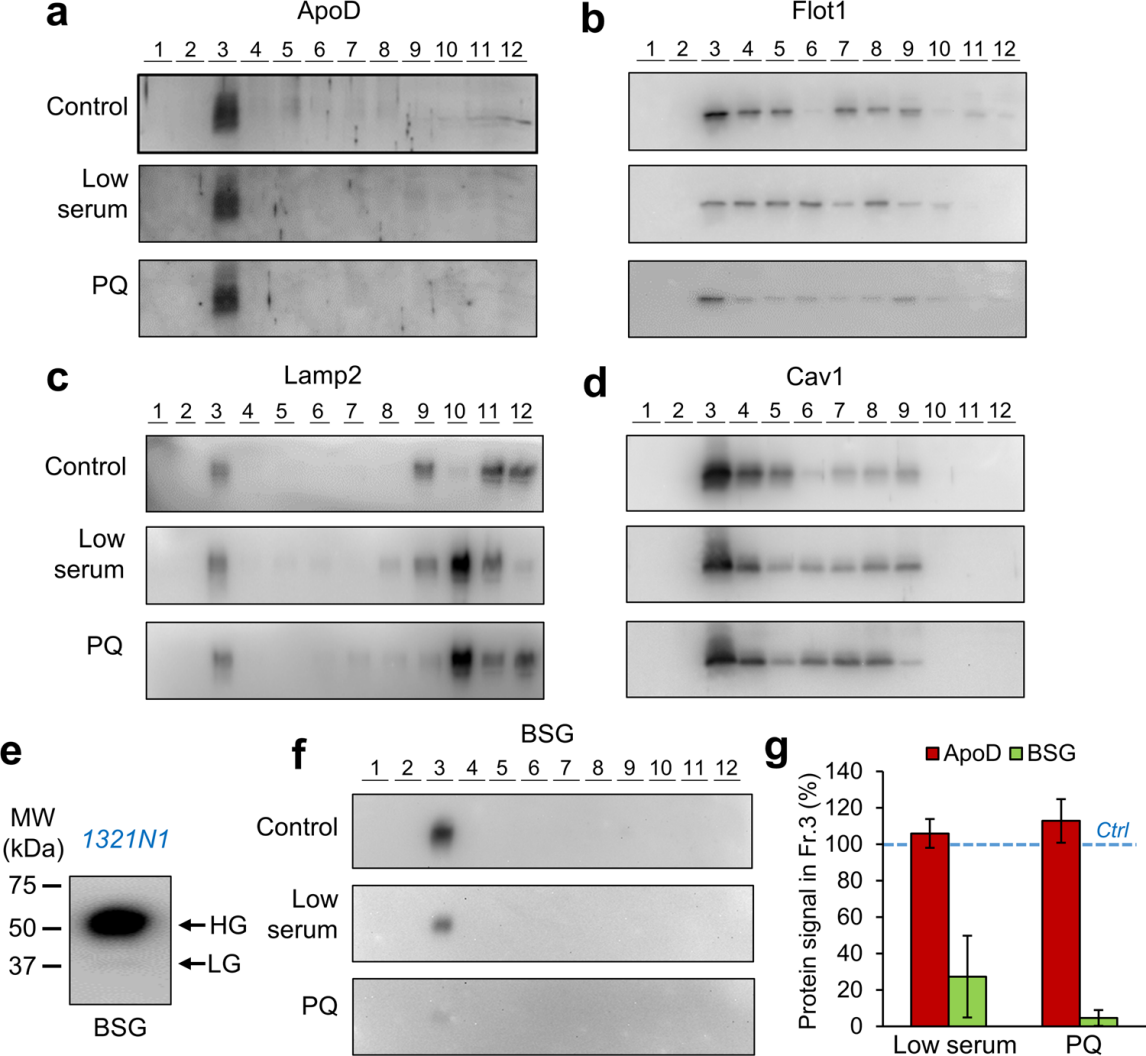
ApoD association with DRMs is maintained in stress conditions. **a**-**d** Immunoblot analysis of ApoD and several membrane-associated proteins in Triton X-114 DRMs of 1321N1 human astrocytoma cells cultured under control, low serum and paraquat (PQ)-driven OS (3-h treatment). No differences in distribution along gradient or net protein amount are observed for ApoD (quantifications shown in Online Resource 1d-f). **e** 1321N1 cells express mostly the high-glycosylated (HG) isoform of Basigin (BSG), but the low-glycosylated (LG) form is also present. **f**-**g** BSG also appears specifically in DRM fraction 3 after sucrose-gradient fractionation of Triton X-114-solubilized 1321N1 membranes. However, its presence decreases upon low-serum or PQ stress. **g** Immunoblot signal for ApoD and BSG in fraction 3 relative to control conditions (*n* = 4 independent experiments, average ± SEM). Only BSG signal is affected by conditions (*p* < 0.01, ANOVA on ranks; no post hoc differences for the LS vs. PQ comparison, Tukey’s test)

ApoD partition into DRMs is stable upon exposure of cells to metabolic (low serum) or OS conditions (PQ treatment, 500 µM, in low-serum media) for 3 h (Fig. 2a). Flot1, Lamp2 and Cav1 are present not only in the ApoD-positive DRM fraction (Fig. 2b-d), but also in fractions with different buoyancy or in the detergent-soluble fractions.

To fully understand the mechanism by which ApoD is able to perform diverse actions on membranes, an important question is whether ApoD-DRM association requires protein–protein interactions. The low-glycosylated form of the membrane glycoprotein BSG has been reported as a ApoD receptor [20]. Low and high-glycosylation forms of BSG (LG-BSG and HG-BSG) are present in different proportions in several cell types [25, 35–37], while HG-BSG is the most abundant isoform in the mouse brain [38]. 1321N1 cells show HG-BSG as the major BSG form, although small amounts of LG-BSG are detected (Fig. 2e).

BSG, as ApoD, is present uniquely in the TX114-resistant high buoyancy fraction (Fig. 2f). However, its enrichment in cell membrane preparations decreases upon acute stress (low-serum or PQ). Quantification of relative presence of ApoD and BSG with respect to control situation (Fig. 2g) shows the clear loss of BSG immunoreactivity, while ApoD is maintained in all conditions in the same DRM preparations (see also ApoD signal profiles quantified in Online Resource 1d). This result is compatible with the reported BSG shedding in extracellular vesicles [39], a process that this result suggests it might be enhanced under stress conditions. However, other explanations based on expression regulation or post-translational modifications are also possible.

Our results demonstrate that while BSG and ApoD are present in membrane biochemical fractions that share common physicochemical properties, their presence in these membranes is differently affected by OS.

### The Subcellular Distribution of Native ApoD in Astrocytic Cells Shows Partial Overlap with BSG

**Fig. 3:**
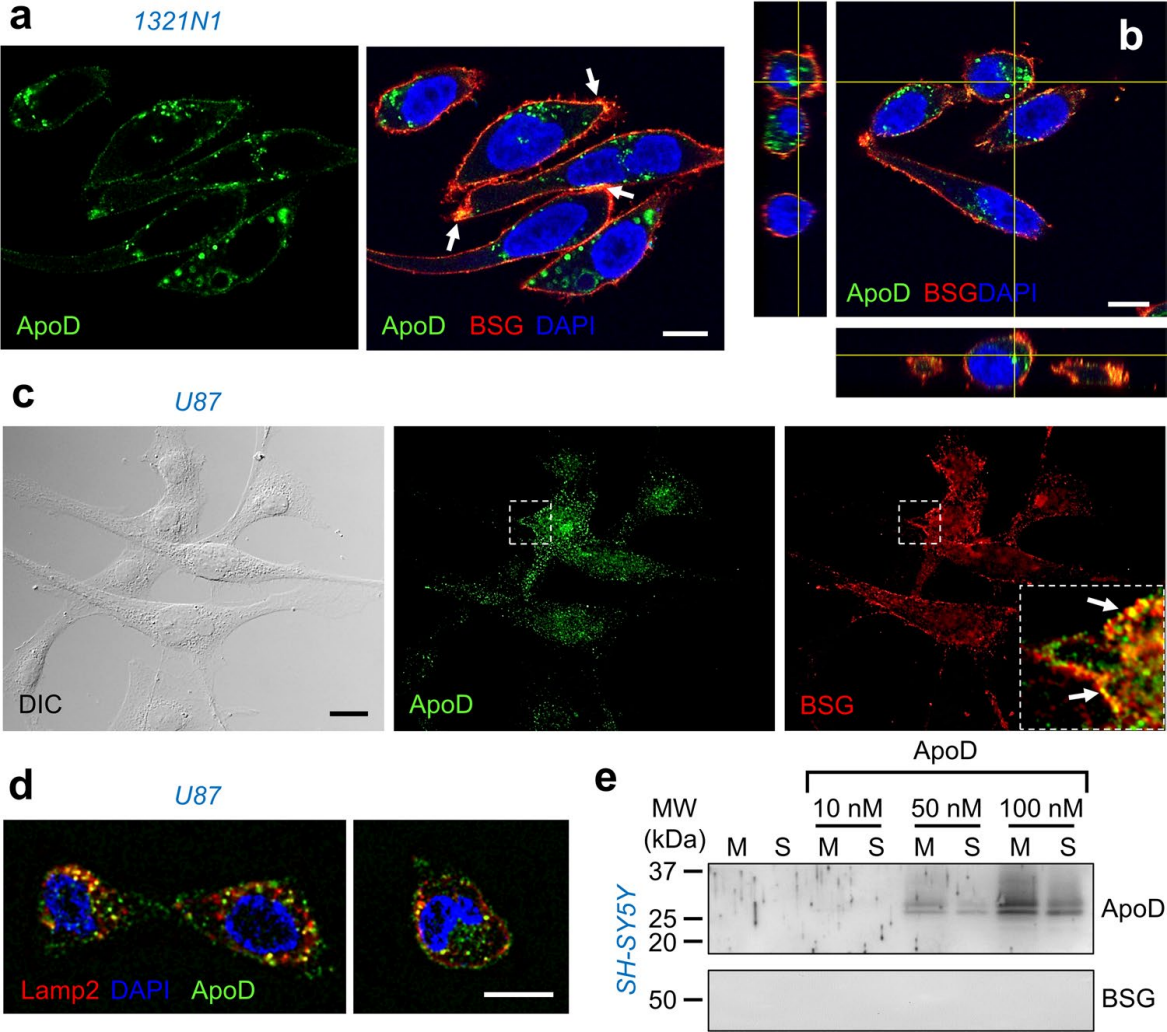
ApoD subcellular localization and membrane interaction in glia and neurons. **a**-**c** ApoD patchily distributes in human astroglial cells and shows overlap on the cell membrane with the evenly located BSG protein. Confocal images of double-labelled native ApoD/BSG under permeabilized conditions show colocalization in 1321N1 cells (arrows in **a**). However, orthogonal views of a Z-stack show ApoD-positive internal organelles with absent BSG labelling (**b**). Similar results are obtained with U87 cells, where ApoD and BSG only co-localize in the plasma membrane (**c**). **d** Representative confocal images showing colocalization of ApoD and the late-endosome-lysosome marker Lamp2 in U87 cells. **e** Exogenous ApoD interacts with SH-SY5Y neuronal membranes in a dose-dependent manner, while no BSG immunoblot signal is observed in these cells. The blot shown was developed in parallel with that of Fig. 2e as positive control. Calibration bars in **a**-**b** and **d**: 10 µm; in **c**: 20 µm

Analysis of the subcellular distribution of BSG and ApoD in 1321N1 astrocytoma cells (Fig. 3a-b) reveals that both proteins co-localize only on the cell surface. ApoD and BSG co-labelling is also observed in U87 glioblastoma cells (Fig. 3c). Thus, two different astrocytic cell lines show only partial overlap of BSG and ApoD immunolocalization. In both cell lines, ApoD has a robust labelling in intracellular organelles from which BSG is absent. As previously described for 1321N1 cells, ApoD traffics to the late-endosome-lysosome compartment (labelled by Lamp2) in U87 cells as well (Fig. 3d and Online Resource 2a). These results might still be compatible with BSG acting as ApoD receptor only at the plasma membrane, a process that could be followed by a BSG-independent ApoD internalization.

### Exogenous ApoD Interacts with Neuronal Membranes in the Absence of BSG

To evaluate whether BSG is a requisite for ApoD binding to cell membranes, we performed in vitro binding assays of hApoD exogenously added to membranes of cells that do not express ApoD, like the neuroblastoma cell line SH-SY5Y. We previously demonstrated that exogenous ApoD is able to protect SH-SY5Y neurons from OS while it is internalized and targeted to lysosomes [12]. As predicted, exogenous ApoD is able to bind to membranes of cells not expressing ApoD in a dose-dependent manner, increasing from 1.1 ± 0.2 to 5.9 ± 0.2 pmols ApoD/mg of membrane proteins (average ± SEM) when exposing membranes to 10 and 100 nM ApoD respectively (Fig. 3e). However, no BSG signal is present in these membrane preparations, as expected for the known slight expression levels of BSG in these cells [40]. These results demonstrate that ApoD-membrane interaction does not require BSG, and they argue against a requirement of BSG for ApoD docking at the cell surface.

Together, these results pose uncertainties on BSG being a mandatory ApoD receptor. This paradox leads us to experimentally test whether BSG is required for ApoD-membrane interactions and intracellular traffic using a Bsg-KO cell line.

### Absence of BSG in Astrocytic Cells Changes Cell Morphology, Proliferation Rate and their Response to Oxidative Stress

**Fig. 4:**
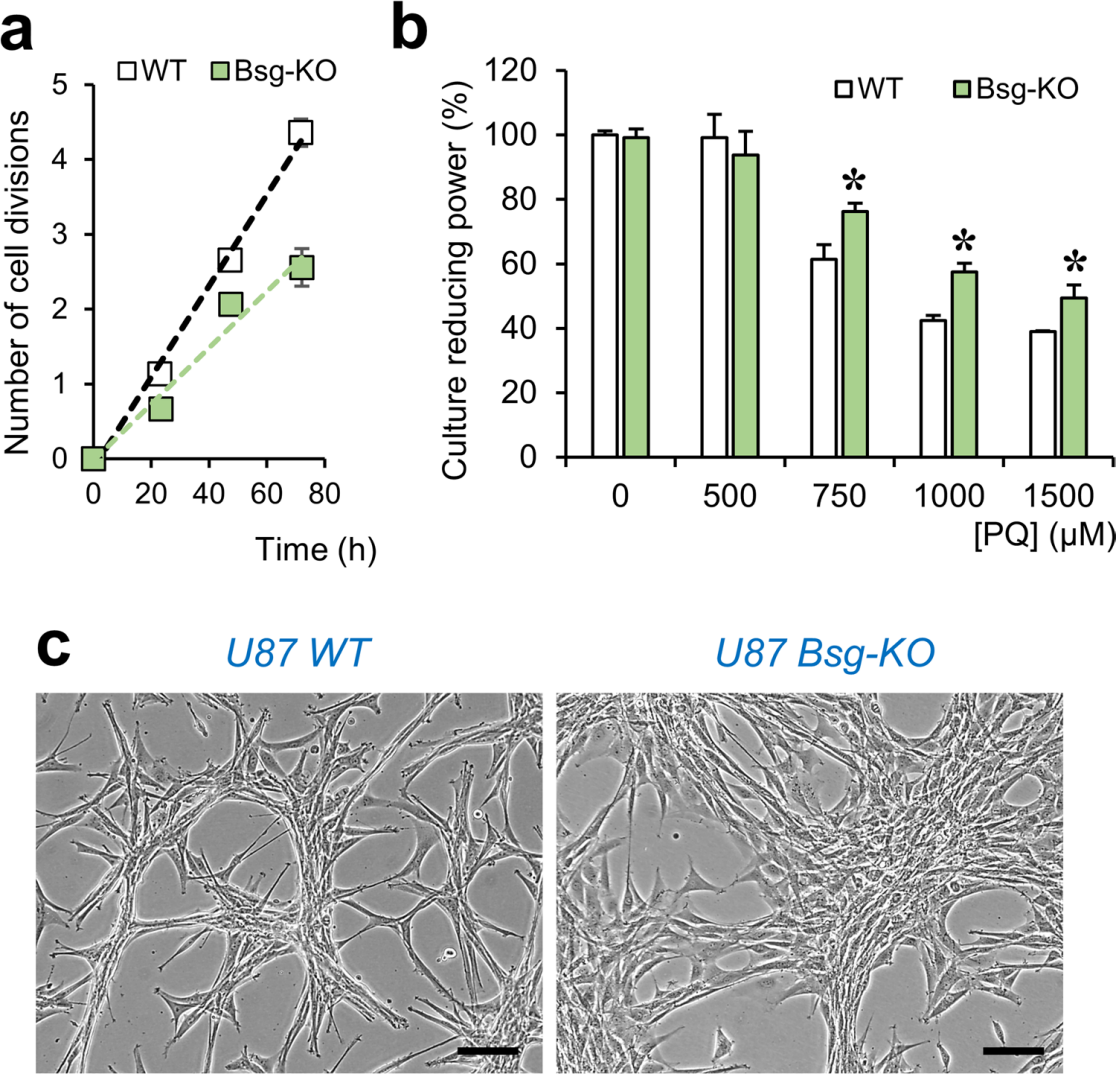
The lack of BSG in glioblastoma U87 cells alters morphology, cell division and OS resistance. **a** Proliferation rate of Bsg-KO cells decreases compared to WT cells (*n* = 3 independent cultures; average ± SEM shown; slope decreases from 0.06 to 0.04 cell divisions/h). **b** Net reducing activity of WT and Bsg-KO cell cultures was measured by the MTT assay. Bsg-KO cells show a higher resistance to increasing doses of PQ for 24 h (ANOVA; genotype *p* < 0.05, PQ dose *p* < 0.001, interaction *p* < 0.001; asterisks represent *p* < 0.05 for genotype comparisons within each PQ dose). **c** Cell morphology is altered by the lack of BSG. Bsg-KO cells loose the characteristic association in threads and knots of U87-WT cells. Calibration bars in **c**: 50 µm

A well-characterized effect of the lack of BSG in glioblastoma U87 cells, mediated by loss-of-function of lactate transporters, is that they switch from glycolytic to oxidative phosphorylation (OXPHOS) metabolism, reducing their division rate with no effect on viability [25]. Our experiments corroborated the lower cell division rate (Fig. 4a) and equal viability (reducing power) of Bsg-KO cell cultures compared with WT U87 cells in control conditions, confirming their bioenergetic plasticity. Interestingly, upon exposure to increasing concentrations of PQ for 24 h, Bsg-KO cells show a higher resistance to OS (Fig. 4b). These cells also show clear morphological differences in culture (Fig. 4c), suggesting differences in cytoskeletal and/or membrane dynamics.

**Fig. 5:**
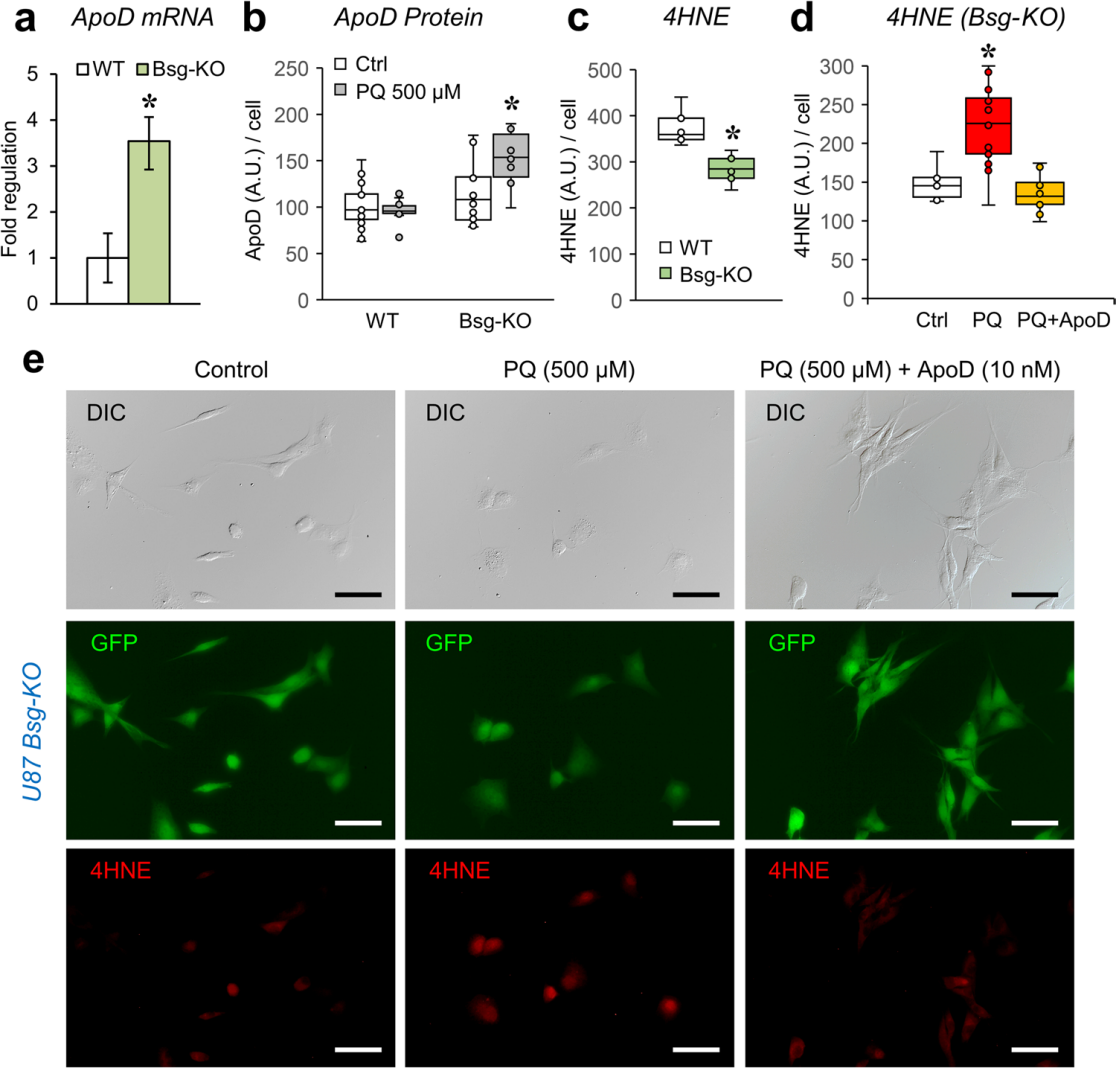
ApoD expression and function upon OS in Bsg-KO astrocytes. **a** qRT-PCR evaluation of ApoD mRNA expression in glioblastoma WT and Bsg-KO cells (Mann–Whitney *U* test, asterisk represents *p* < 0.01). **b** ApoD protein expression (fluorescence immunocytochemistry quantification) of WT and Bsg-KO cells in control and OS conditions (500 µM PQ exposure, 24 h) (ANOVA; genotype *p* < 0.001, condition *p* < 0.05, interaction *p* = 0.01; asterisk represents *p* < 0.001 for genotype comparisons within each Bsg genotype). **c** 4HNE levels in WT and Bsg-KO cells measured by fluorescence immunocytochemistry quantification (Student *T* test, asterisk represents *p* < 0.001). **d**-**e** 4HNE levels in Bsg-KO cells exposed to OS (500 µM PQ) and the antioxidant effect of exogenous hApoD simultaneous addition (10 nM) for 2 h. Quantification of 4HNE-fluorescence (**d**) and representative microscopy fields (**e**). Bsg-KO cells express cytoplasmic GFP. ANOVA on ranks in **d**, asterisk represents *p* < 0.05 in Dunn’s test for PQ vs. the other two conditions. A.U.: arbitrary units. Calibration bars in **e**: 50 µm

Their resilience upon OS might have relevant influences on ApoD biology: An over-expression of ApoD upon PQ is predicted, as described for 1321N1 cells or mouse primary astrocytes [13]. We therefore quantitated ApoD mRNA expression (Fig. 5a) and protein expression (Fig. 5b) upon PQ in Bsg-KO and WT U87 cells. We used a dose (500 µM PQ) where the reducing power of the cell culture is still not compromised (Fig. 4b), avoiding confounding effects. While ApoD mRNA is maintained at very low levels in WT cells, it has significantly higher levels in Bsg-KO cells. Interestingly, ApoD shows similar basal protein levels in WT and Bsg-KO cells, but it is boosted upon PQ treatment in Bsg-KO only (Fig. 5b). These results suggest that Bsg-KO cells are primed with high mRNA levels of the endogenous protector ApoD. Protein accumulation is subsequently promoted upon sub-lethal PQ exposure.

### ApoD Does Not Require BSG to Perform its Antioxidant Function

ApoD mRNA and protein expression response correlate with a better resilience of Bsg-KO cells upon high doses of PQ. This suggests not only that there is an ApoD expression induction downstream to BSG-loss, but also that a lack of BSG might not interfere with ApoD protective function. To measure lipid peroxidation derivatives was therefore relevant. Lack of BSG results in lower basal levels of 4HNE (Fig. 5c). This observation is compatible with a higher basal activity of antioxidant mechanisms (ApoD among others), not unexpected in these OXPHOS-dependent cells. Moreover, the addition of exogenous native ApoD to PQ-treated Bsg-KO cells is able to revert the PQ-induced increase in 4HNE (Fig. 5d-e), demonstrating that the ApoD lipid-antioxidant function [9] does not require BSG.

### Exogenous ApoD Can Be Internalized by Astrocytic Cells in the Absence of BSG

We have previously demonstrated that ApoD internalization and stable presence in acidic organelles are required to fulfil its protective function in both ApoD-expressing astroglial and non-expressing neuronal cells [12]. Since U87 cells are no exception for ApoD intracellular location (Fig. 3d and Online Resource 2a), we therefore tested whether BSG is required for ApoD internalization in these cells. We used exogenous hApoD fluorescently labelled so that it can be visualized after internalization in both WT and Bsg-KO U87 cells.

**Fig. 6:**
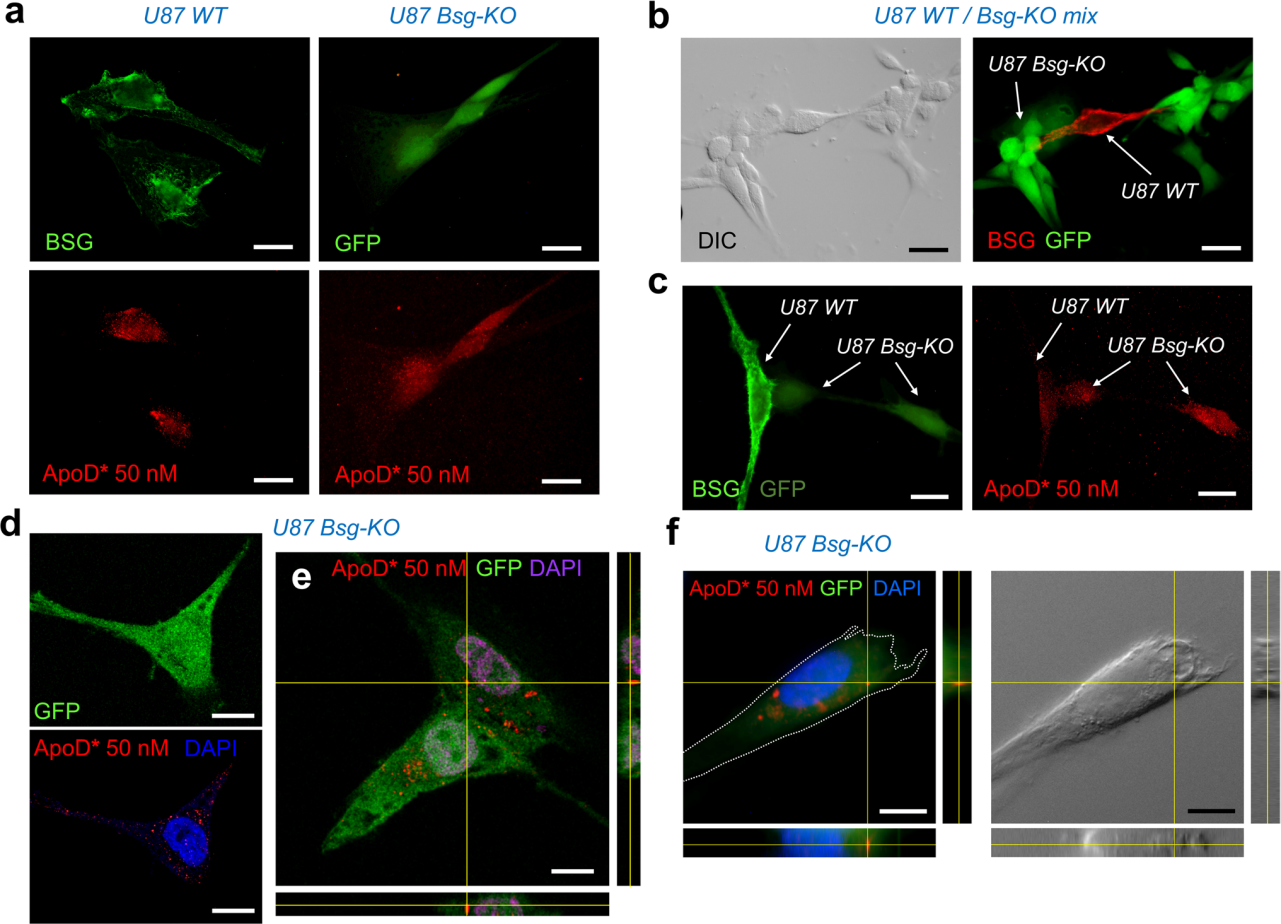
ApoD internalization is independent of BSG. **a** Exogenously added (50 nM, 3-h exposure) fluorescently labelled ApoD (ApoD*) is detected inside both WT and Bsg-KO cells. ApoD is directly detected in the 594-nm channel by fluorescence microscopy. BSG, monitored with an Alexa-488 secondary antibody, is detected in membranes of WT cells only. Bsg-KO express a cytoplasmic form of GFP. **b** Mixed cultures (WT:Bsg-KO, 1:10 ratio) show expression of BSG (Alexa-594 secondary antibody) only in GFP-negative (Bsg-KO) cells. **c** ApoD addition to mixed cultures evidences simultaneous internalization independent on cell genotype. BSG membrane labelling (green surface), cytoplasmic GFP labelling (diffuse intracellular) and ApoD (red) are shown. **d**-**f** Internalized ApoD*-positive vesicles are evidenced in Bsg-KO U87 cells by single confocal optical sections (**d**), by confocal Z-stacks with orthogonal views (**e**), as well as by deconvolution of fluorescence microscopy Z-stacks (**f**). Calibration bars in **a**-**b**: 20 µm; in **c**-**e**: 10 µm

ApoD is detected in internal organelles of both WT and Bsg-KO cells after 3-h exposure to fluorescently labelled hApoD (Fig. 6a). To visualize the internalization process in the same culture, we spiked a Bsg-KO culture with WT cells at a 1:10 ratio (Fig. 6b), where a segregation of BSG membrane labelling in WT cells and GFP presence in Bsg-KO cells is evident. In these conditions, ApoD is internalized in both Bsg-KO and WT cells (Fig. 6c). Analyses of single confocal sections (Fig. 6d), confocal Z-stacks (Fig. 6e) or deconvolved images from fluorescence microscopy Z-stacks (Fig. 6f) clearly show ApoD inside the cells. Online Resource 2 b-c show negative controls (used for ApoD-labelled 594 nm detection channel) performed in parallel to labelled ApoD internalization. Our results unambiguously demonstrate that internalization of ApoD does not require BSG.

### ApoD and BSG Are Segregated in DRMs with Different Physicochemical Properties

BSG and ApoD are present in DRMs with similar up-thrust in our sucrose step gradients (Fig. 2a, f). This result therefore suggests that there might be a mixed pool of DRMs in this fraction. Sub-fractionation of TX114-DRMs with different buoyancy properties can be achieved by four, five and six-step sucrose gradients in 1321N1 cells, where densities lower than 35% sucrose were explored.

**Fig. 7:**
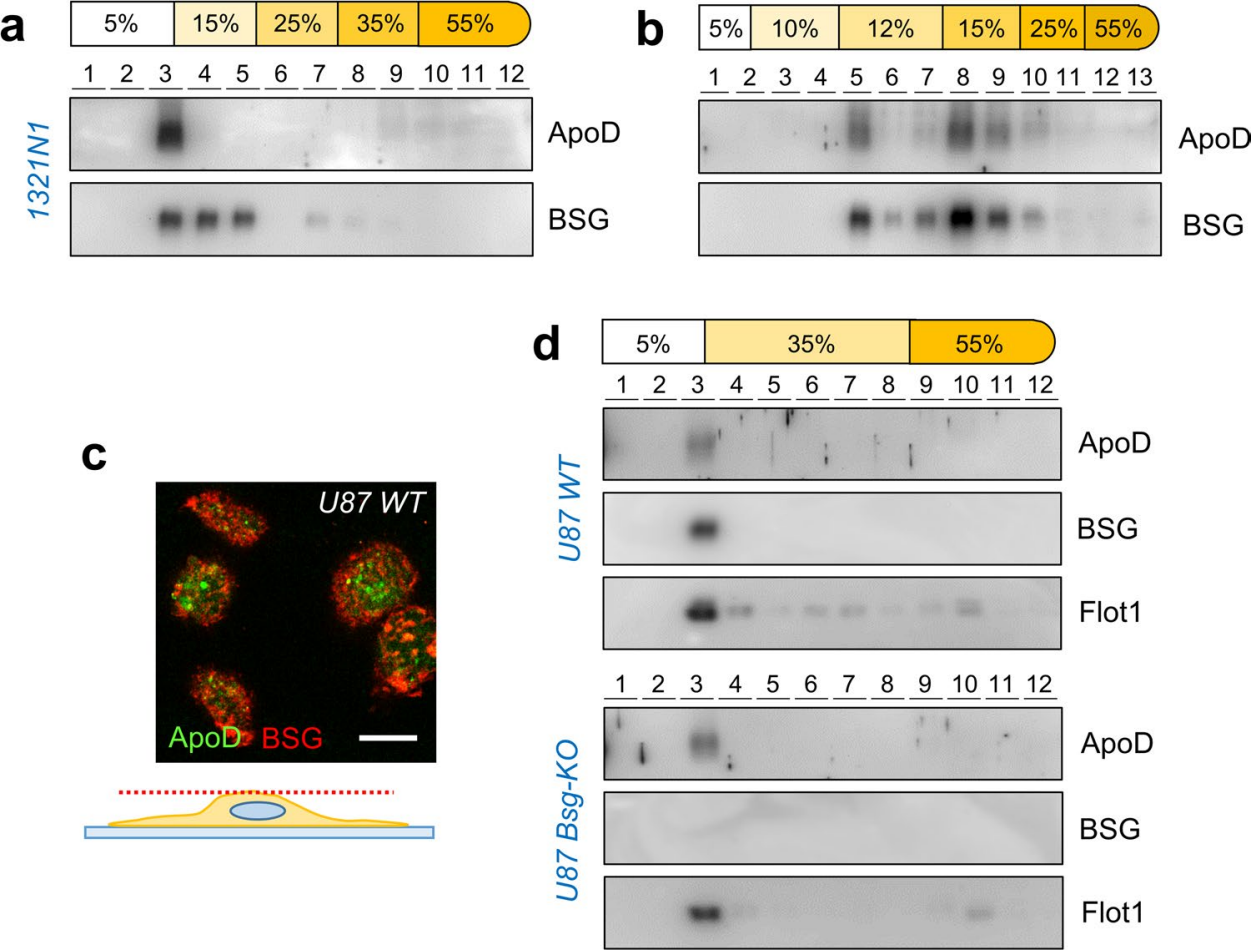
ApoD-DRM interaction does not require BSG, and both proteins are present in membrane domains with different physicochemical properties. **a**-**b** Immunoblot analyses of Triton X-114-solubilized membrane preparations of 1321N1 glial cells fractionated in different discontinuous sucrose densities. Representative immunoblots are shown (*n* = 4 independent multistep-gradient experiments, two of each type). Human ApoD separates from BSG distribution in sucrose step gradients of five (**a**) and six (**b**) phases. **c** Single confocal section of the cell surface detecting native ApoD and BSG by double immunocytochemistry in U87 WT cells. No significant colocalization is observed. **d** Human ApoD is specifically detected in Triton X-114 DRMs (fraction 3) in both WT U87 and Bsg-KO cells, co-fractionating with Flot1. BSG is obviously not present in Bsg-KO DRMs. Representative immunoblots are shown (*n* = 3 independent experiments). Calibration bar in **c**: 10 µm

When DRMs are allowed to migrate on 15% sucrose, ApoD-positive DRMs show a higher buoyancy, and this partition pattern coincides only partially with a more dispersed BSG distribution in DRMs with lower buoyancy (Fig. 7a). The membrane sub-fractions containing BSG and not ApoD have clearly distinguishable textures (Online Resource 3a) within the 15% sucrose phase.

When even lower sucrose densities are tested (Fig. 7b), ApoD co-migrates with two major subtypes of DRMs with neutral buoyancy at two different sucrose density steps: 12% and 15%. Again, BSG co-migrates with DRMs with a wider range of flotation properties and textures (Online Resource 3b).

Characterization of the different types of TX114-DRMs observed in the 6-step gradients (Online Resource 3c) shows that Cav1 and Lamp2 are differentially enriched in the two major ApoD-positive DRM fractions (#5 and #8), being Cav1 more abundant in the higher buoyancy fraction (#5) and Lamp2 in the one floating at 15% sucrose (#8). This result is coherent with the two major locations of ApoD in the cell (plasma membrane and lysosomes) and suggests that, at those locations, ApoD might be associated with DRMs with different physicochemical properties, worth of further analytical studies.

Finally, analysis of the cell surface in confocal sections of U87-WT cells (Fig. 7c) clearly shows that ApoD and BSG locate at mostly non-overlapping membrane patches, coherent with their different partition pattern in multistep sucrose gradients.

### ApoD-DRM Interaction Does Not Require BSG

We then proceeded to test whether ApoD-DRM interaction requires BSG (Fig. 7d). The specific fractionation of ApoD in TX114 DRMs with buoyancy over 35% sucrose does not depend on BSG presence in astrocytic membranes, since it is maintained in Bsg-KO U87 cells.

In summary, our results demonstrate that: (1) ApoD and BSG distribute in membrane domains with different physicochemical properties; (2) BSG mostly locates to the plasma membrane, while ApoD is observed in both plasma membrane and intracellular organelles/compartments; (3) neither ApoD internalization nor ApoD-membrane interaction requires BSG; and (4) ApoD lipid-antioxidant function does not require BSG.

## Discussion

This work demonstrates that ApoD is a natural resident of cellular membranes in glial cells. ApoD-membrane association is robust and maintained throughout the process of membrane isolation and fractionation into detergent-soluble/resistant subdomains. ApoD is detected neither in dense organelles pelleted at low centrifugal force in the process, nor in the soluble fraction remaining after ultracentrifugation of membranes. Furthermore, we demonstrate that ApoD can also interact in vitro with membranes of neuronal cells that do not have endogenous ApoD expression, demonstrating that ApoD-membrane association is a basic biochemical property of this lipocalin. The existence of ApoD insect homologues anchored to cell membranes through glycosyl-phosphatidylinositol moieties [41, 42] also supports that membrane interaction is an ancestral property in this family.

Whether this interaction is mediated by a membrane protein receptor is a relevant question. We explored the membrane glycoprotein BSG, a reported ApoD receptor candidate. Its low-glycosylated form (LG-BSG) was proposed to mediate ApoD internalization in SH-SY5Y cells overexpressing BSG [20]. However, non-transfected SH-SY5Y cells with undetectable levels of BSG also appear in that work to internalize exogenously added ApoD, as was previously reported for SH-SY5Y [12, 18]. Here, we demonstrate a dose-dependent in vitro interaction of ApoD with SH-SY5Y membranes in the absence of native BSG expression, in agreement with the ability of ApoD to exert its function on neurite differentiation in SH-SY5Y cells [26]. The low proportion of LG-BSG vs. HG-BSG reported in both mouse brain [38] and the astrocytic line 1321N1, tested in this work, add doubts to its requirement as ApoD receptor. Moreover, brain endothelial cells, with significant levels of LG-BSG expression, are able to transcytose ApoD in a BSG-independent manner [38]. Finally, the physical interaction of ApoD and specific subdomains in plasma and lysosomal membranes of glial and neuronal cells do not require BSG, although the two proteins might share some of these membrane compartments.

The differential behaviour of ApoD and BSG upon OS is worth further analysis, since it brings interesting scenarios of stimulus-triggered protein sorting mechanisms. Upon acute stress, BSG and ApoD might follow different routes to either get internalized (ApoD) [12] or exported into plasma membrane-derived extracellular vesicles (BSG) [39, 43].

Curiously, our experiments also demonstrate that ApoD retains its membrane-associated functional niche not only under OS conditions, but also in cells with different metabolic strategies, relying on glycolysis (Bsg-WT) or on mitochondrial oxidative activity (Bsg-KO) [25]. This property would facilitate ApoD beneficial actions on the membranes of different cell types in the brain, independently of their metabolism. Thus, whether the glia-neuron metabolic coupling [44] is working properly in healthy conditions, or it is disrupted in disease, ApoD should still be able to control the redox state of their membranes.

The known functional consequences of ApoD presence in the lysosomal compartment, so important for glial and neuronal cells [12, 15], can now be visualized as a direct effect on lysosomal membranes, preventing their permeabilization, conditioning traffic to-and-from the plasma membrane and the activity of enzymes relevant to membrane composition. These functions require native ApoD, since bacterial recombinant ApoD, where glycosylation and hydrophobic surface patches are not present, cannot be retained in the lysosome [15].

Our data demonstrate for the first time that ApoD is confined to DRMs, particularly to fractions enriched in plasma and lysosomal membranes resistant to TX114 solubilization, and that ApoD-DRM association is maintained under OS conditions. They provide the basic requirements for ApoD to exert its antioxidant activity on membranes. Such a mechanism would explain the known ability of ApoD in keeping lipid peroxidation levels under control (demonstrated in mammals at organism, tissue and cellular levels) [6, 12, 13, 16, 45], and preserved even when human ApoD function was tested in insect or plant model organisms [46, 47]. Our findings are also coherent with the known antioxidant properties of ApoD-containing HDL_3_ lipoparticles ([48], reviewed by [8]), and support the protective roles of ApoD within and outside the nervous system. ApoD-positive DRMs can be further fractionated by their different buoyancy, indicating the presence of ApoD in membrane subdomains with different physicochemical properties, and possibly present in different subcellular locations. Compared to classic DRM markers, like Flot1 or Cav1, ApoD is revealed as a very specific and useful marker of subtypes of DRMs worth to study.

Identification of ApoD site of action at particular membrane subdomains in neuronal and glial cell membranes should therefore provide the appropriate research focus to further dissect its neuroprotective mechanism and its contribution to membrane maintenance and repair processes, which are of special relevance for a functional nervous system.

## Supplementary Information

Below is the link to the electronic supplementary material.

Online Resource 1 Characterization of TX114-DRMs from astrocytic membranes. Gene ontology terms significantly enriched (FDR < 0.005) in proteins found in TX114-DRMs of mouse primary astrocytes (listed in Online Resource 4). The number of proteins for each GO category is shown in italics. **b** Venn diagram of DRM mouse proteins found in our work and those annotated in RaftProtV2 database (listed in Online Resource 4). **c** List of proteins from TX114-DRMs of mouse primary astrocytes ascribed to membrane raft or lysosome GO terms. **d**–**f** Immunoblot signal profiles along discontinuous sucrose gradients after TX114 solubilisation of membranes from human astroglioma 1321N1 cells. Signal in each fraction normalized to total signal in the blot. **d** ApoD, n=5 independent experiments per condition (C, control; LS, low serum medium for 3 hours; PQ, 500 μM paraquat in low serum medium for 3 hours). **e** Flot1, n=4 independent experiments per condition. **f** Cav1, n=3 independent experiments per condition (PNG 48 kb)

High resolution image (TIF 942 KB)

Online Resource 2 Additional microscopy experiments examples and controls. **a** Representative confocal images showing colocalization of ApoD and the late-endosome-lysosome marker Lamp2 in U87 WT cells. **b**–**c** Immunofluorescence microscopy controls for the detection of exogenous fluorescently labelled ApoD (ApoD*). Bsg-KO cells express the cytoplasmic GFP marker while no BSG is immunodetected (Alexa-488 secondary antibody). Emission at 594 nm is not present in WT or Bsg-KO U87 cells in control conditions (**b**). Labelled ApoD* signal internalized by WT cells (**c**)  is evidenced in parallel to cultures shown in **b**. Calibration bars in a: 10 μm; in b–c: 20 μm (PNG 48 kb)

High resolution image (TIF 4.13 MB)

Online Resource 3 Sub-fractionation of TX114-DRMs of different buoyancy in 1321N1 cells. **a** Image and schematic representation of the visible floating fractions in a 5-step gradient experiment. Inset shows fraction 4, where two distinct but difficult to separate DRM types are evident. **b** Image and schematic representation of the visible floating fractions in a 6-step gradient experiment. Inset shows fraction 6, with a distinctive globular texture. **c** Immunoblot analyses of ApoD, BSG and several membrane proteins in TX114-solubilized membrane preparations fractionated in 13 samples from a six-phase discontinuous sucrose density centrifugation as the one shown in **b** (PNG 48 kb)

High resolution image (TIF 5.56 MB)

Online Resource 4 Analysis of proteins identified by mass spectrometry in TX114-DRMs of mouse primary astrocytes and comparisons with mouse proteins annotated in the RaftProtV2 database (XLSX 71.7 KB)

## Data Availability

All data generated and analysed during this study are included in this published article and its Online Resources files.
