# Supplementary figures and images for: The Neuroprotective Lipocalin Apolipoprotein D Stably Interacts with Specific Subtypes of Detergent-Resistant Membrane Domains in a Basigin-Independent Manner
Source: Mol Neurobiol. 2022 Apr 22;59(7):4015–29. doi: 10.1007/s12035-022-02829-z (PMC9167181; doi:10.1007/s12035-022-02829-z)

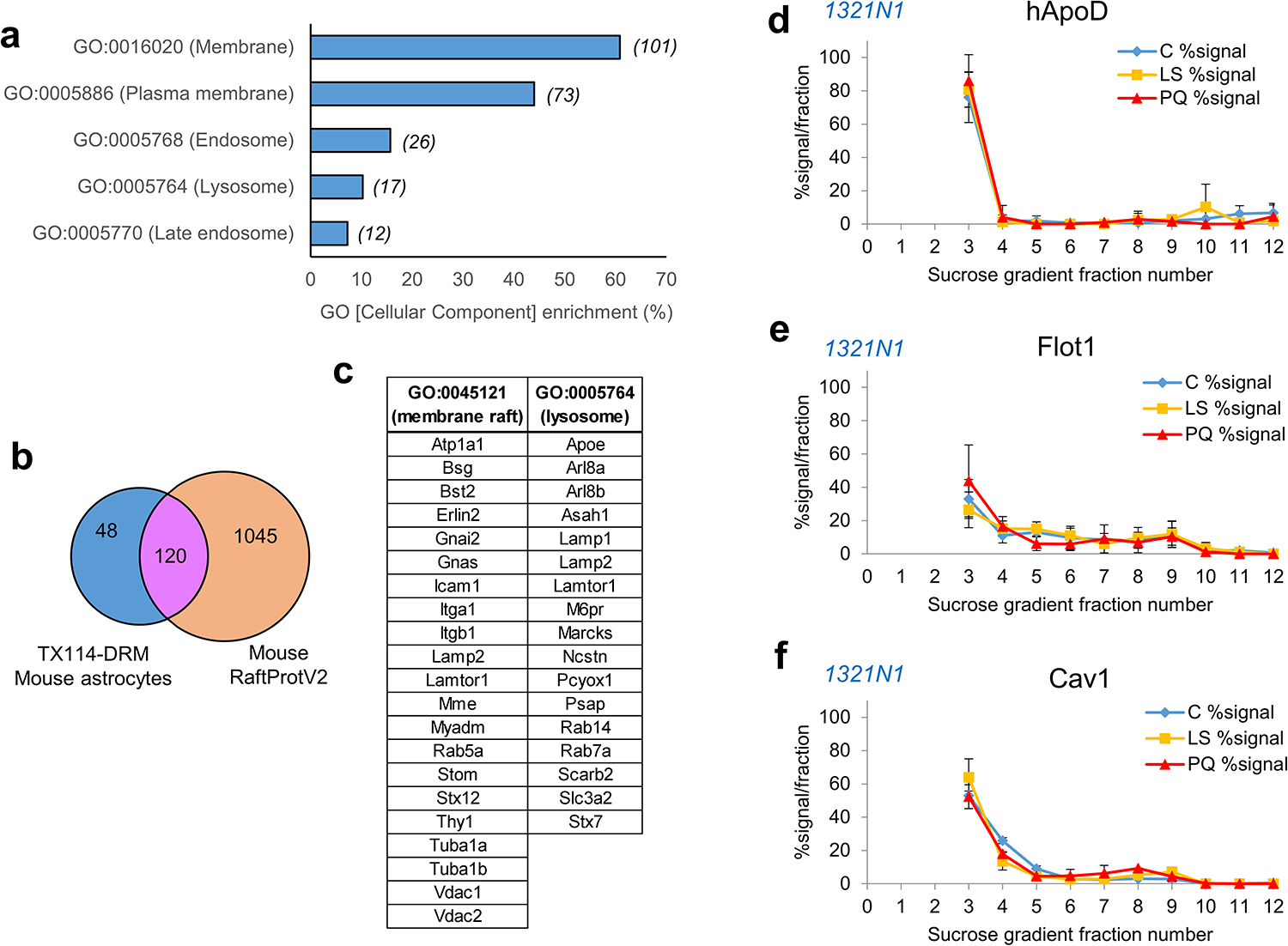

Supplement: Supplementary file 1 — Characterization of TX114-DRMs from astrocytic membranes. Gene ontology terms significantly enriched (FDR < 0.005) in proteins found in TX114-DRMs of mouse primary astrocytes (listed in Online Resource 4). The number of proteins for each GO category is shown in italics. b Venn diagram of DRM mouse proteins found in our work and those annotated in RaftProtV2 database (listed in Online Resource 4). c List of proteins from TX114-DRMs of mouse primary astrocytes ascribed to membrane raft or lysosome GO terms. d–f Immunoblot signal profiles along discontinuous sucrose gradients after TX114 solubilisation of membranes from human astroglioma 1321N1 cells. Signal in each fraction normalized to total signal in the blot. d ApoD, n=5 independent experiments per condition (C, control; LS, low serum medium for 3 hours; PQ, 500 μM paraquat in low serum medium for 3 hours). e Flot1, n=4 independent experiments per condition. f Cav1, n=3 independent experiments per condition (PNG 48 kb) [file 12035_2022_2829_Fig8_ESM.png]

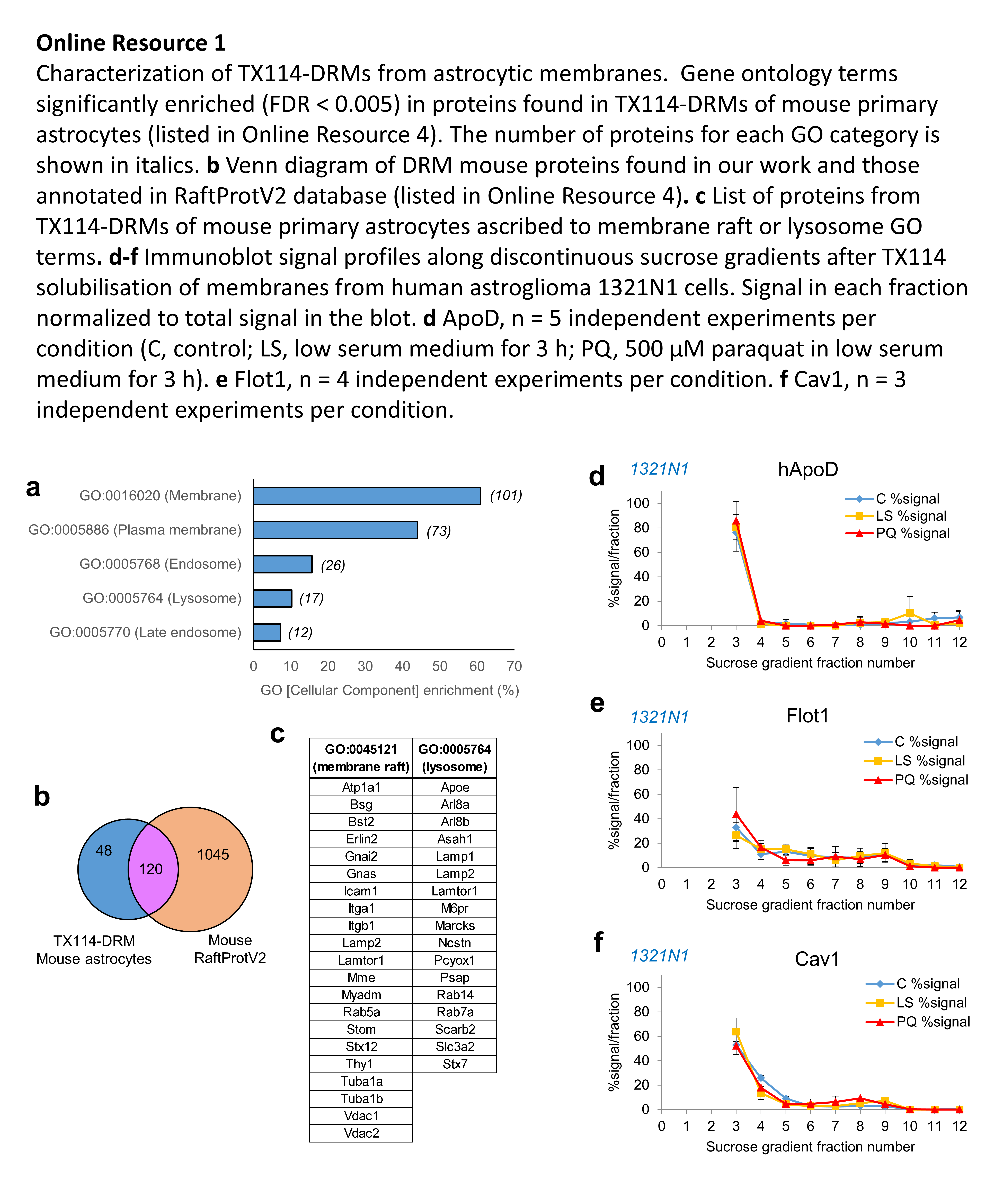

Supplement: Supplementary file 2 — High resolution image (TIF 942 KB) [file 12035_2022_2829_MOESM1_ESM.tif]

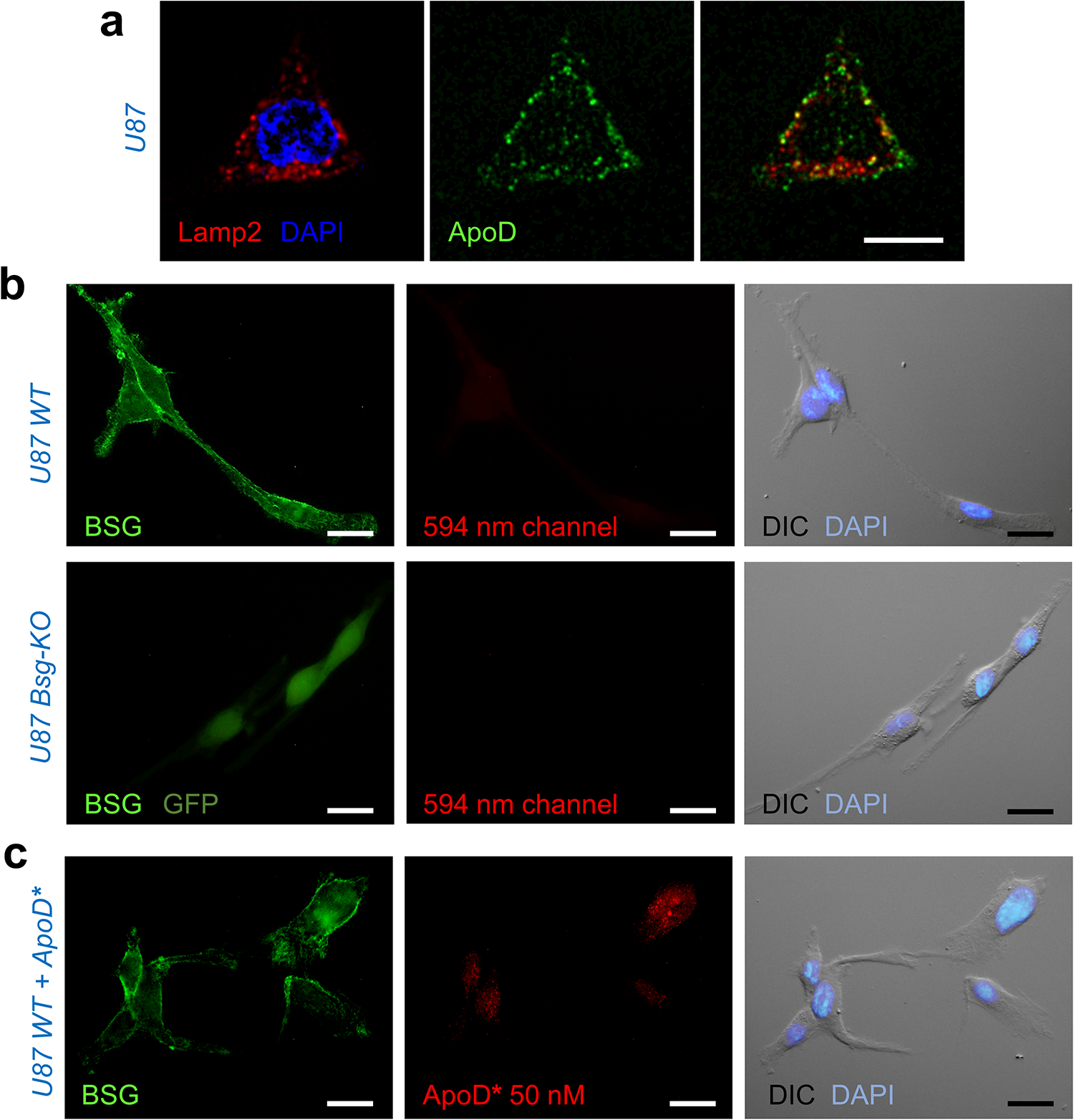

Supplement: Supplementary file 3 — Additional microscopy experiments examples and controls. a Representative confocal images showing colocalization of ApoD and the late-endosome-lysosome marker Lamp2 in U87 WT cells. b–c Immunofluorescence microscopy controls for the detection of exogenous fluorescently labelled ApoD (ApoD*). Bsg-KO cells express the cytoplasmic GFP marker while no BSG is immunodetected (Alexa-488 secondary antibody). Emission at 594 nm is not present in WT or Bsg-KO U87 cells in control conditions (b). Labelled ApoD* signal internalized by WT cells (c) is evidenced in parallel to cultures shown in b. Calibration bars in a: 10 μm; in b–c: 20 μm (PNG 48 kb) [file 12035_2022_2829_Fig9_ESM.png]

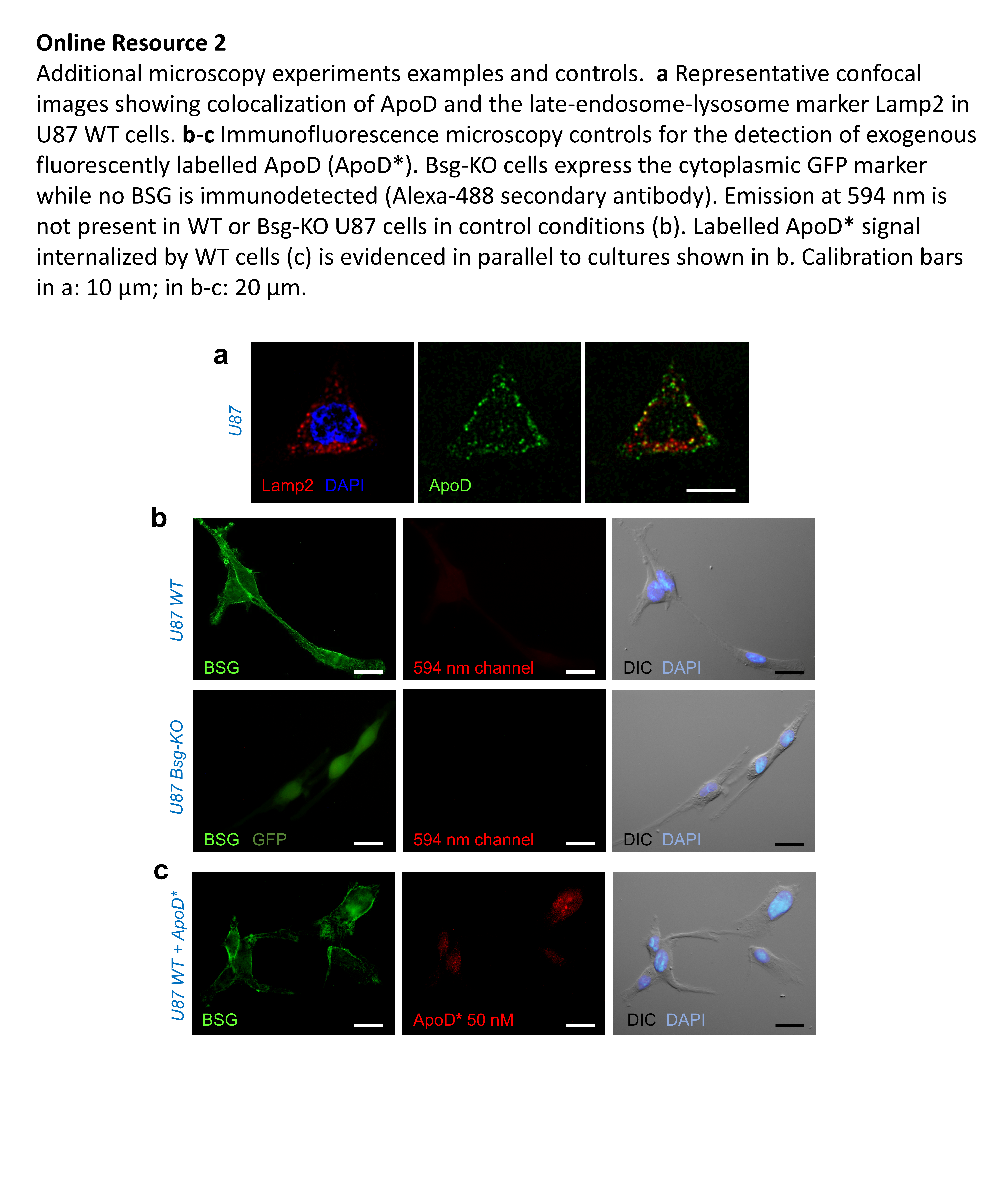

Supplement: Supplementary file 4 — High resolution image (TIF 4.13 MB) [file 12035_2022_2829_MOESM2_ESM.tif]

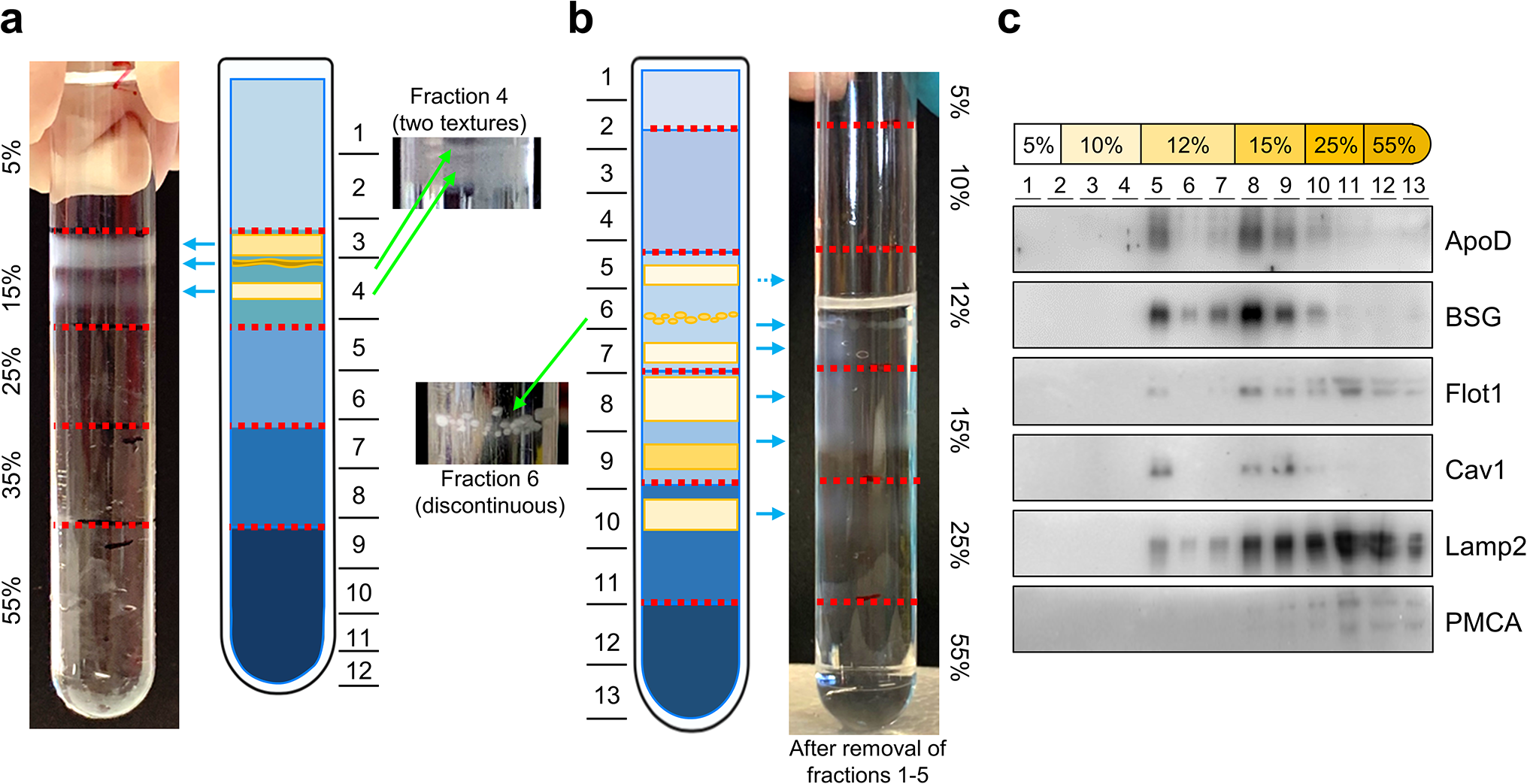

Supplement: Supplementary file 5 — Sub-fractionation of TX114-DRMs of different buoyancy in 1321N1 cells. a Image and schematic representation of the visible floating fractions in a 5-step gradient experiment. Inset shows fraction 4, where two distinct but difficult to separate DRM types are evident. b Image and schematic representation of the visible floating fractions in a 6-step gradient experiment. Inset shows fraction 6, with a distinctive globular texture. c Immunoblot analyses of ApoD, BSG and several membrane proteins in TX114-solubilized membrane preparations fractionated in 13 samples from a six-phase discontinuous sucrose density centrifugation as the one shown in b (PNG 48 kb) [file 12035_2022_2829_Fig10_ESM.png]

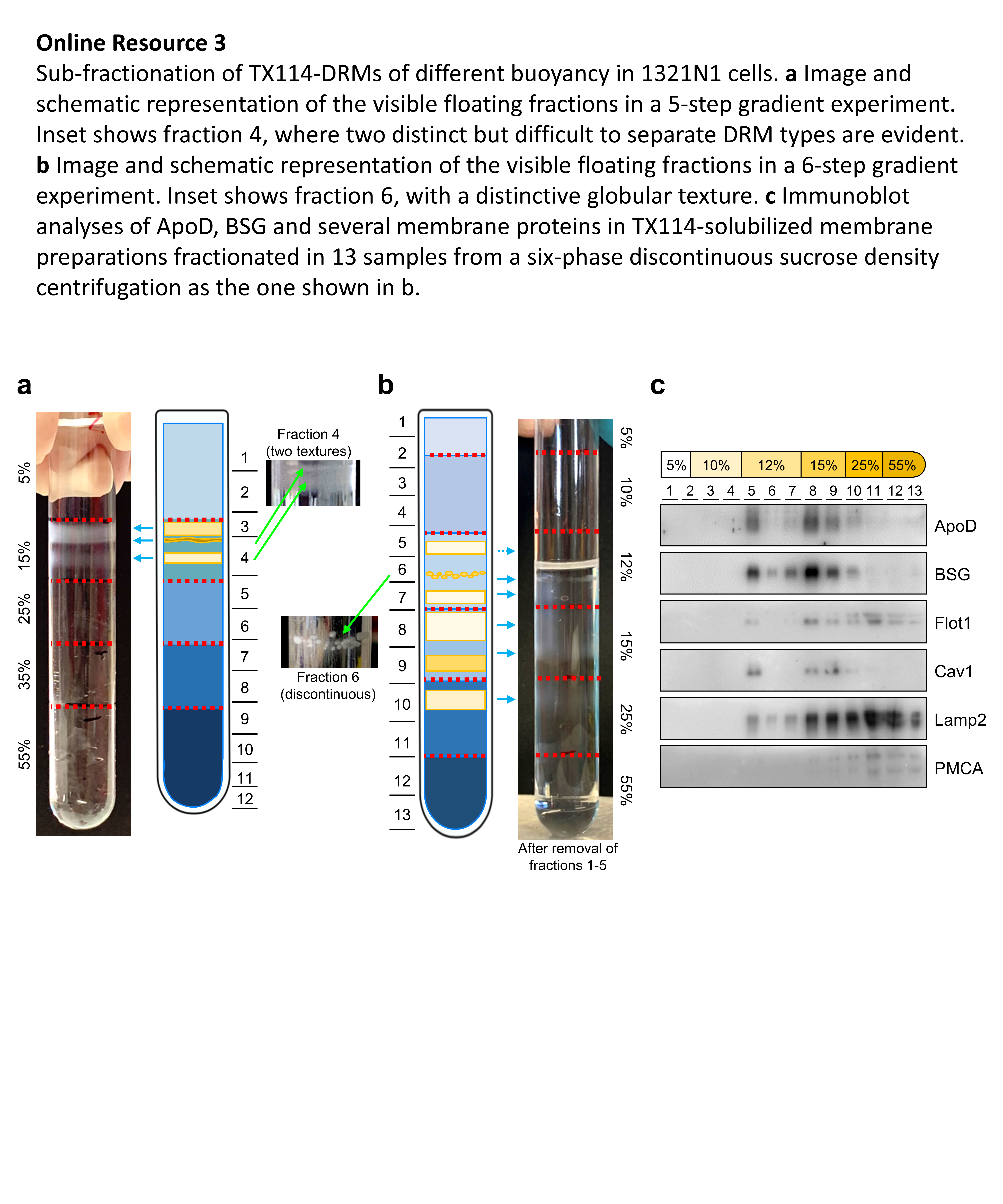

Supplement: Supplementary file 6 — High resolution image (TIF 5.56 MB) [file 12035_2022_2829_MOESM3_ESM.tif]
